# Probiotics in the Management of Mental and Gastrointestinal Post-COVID Symptomes

**DOI:** 10.3390/jcm11175155

**Published:** 2022-08-31

**Authors:** Igor Łoniewski, Karolina Skonieczna-Żydecka, Joanna Sołek-Pastuszka, Wojciech Marlicz

**Affiliations:** 1 Department of Biochemical Science, Pomeranian Medical University in Szczecin, 71-460 Szczecin, Poland; 2 Department of Anesthesiology and Intensive Therapy, Pomeranian Medical University, 70-204 Szczecin, Poland; 3 Department of Gastroenterology, Pomeranian Medical University, 70-204 Szczecin, Poland

**Keywords:** COVID-19, probiotics, microbiota, gut barrier, post-COVID-19 syndrome

## Abstract

Patients with “post-COVID” syndrome manifest with a variety of signs and symptoms that continue/develop after acute COVID-19. Among the most common are gastrointestinal (GI) and mental symptoms. The reason for symptom occurrence lies in the SARS-CoV-2 capability of binding to exact receptors, among other angiotensin converting enzyme 2 (ACE2) receptors in gastrointestinal lining and neuropilin-1 (NRP-1) in the nervous system, which leads to loss of gastrointestinal and blood-brain barriers integrity and function. The data are mounting that SARS-CoV-2 can trigger systemic inflammation and lead to disruption of gut-brain axis (GBA) and the development of disorders of gut brain interaction (DGBIs). Functional dyspepsia (FD) and irritable bowel syndrome (IBS) are the most common DGBIs syndromes. On the other hand, emotional disorders have also been demonstrated as DGBIs. Currently, there are no official recommendations or recommended procedures for the use of probiotics in patients with COVID-19. However, it can be assumed that many doctors, pharmacists, and patients will want to use a probiotic in the treatment of this disease. In such cases, strains with documented activity should be used. There is a constant need to plan and conduct new trials on the role of probiotics and verify their clinical efficacy for counteracting the negative consequences of COVID-19 pandemic. Quality control is another important but often neglected aspect in trials utilizing probiotics in various clinical entities. It determines the safety and efficacy of probiotics, which is of utmost importance in patients with post-acute COVID-19 syndrome.

## 1. Introduction

After the first cases of SARS-CoV-2 virus contamination in Wuhan, Hubei Province, China, in December 2019 and the announcement of World Health Organization (WHO) declaring a global pandemic on 11 March 2020, COVID-19 infected over 589 million people around the world, caused over 6,400,000 deaths on 13 August 2022 [1]. Due to the genetic evolution of the virus, up to date five different variants of concern have been identified, namely, Alpha (B.1.1.7), Beta (B.1.351), Gamma (P.1), Delta (B.1.617.2), and Omicron (B.1.1.529) [2].

Respiratory presentation of COVID-19 is predominant; however, a set of other symptoms, including gastrointestinal ones along with hepatic malfunctions, might precede full symptomatic occurrence or co-occur with respiratory infection [3,4]. Most COVID-19 patients are oligosymptomatic with scarce or discreet symptoms. In selected individuals, SARS-CoV-2 damage may be intensified due to an inadequate immune response [5] leading to whole body failure [6]. Apart from acute COVID-19 symptoms, of relevance are adverse events associated with long COVID-19 disease. In fact, in persons who experienced mild symptoms of COVID-19, as well as survivors, post-acute COVID-19 syndrome (PACS) might occur [7] as a set of persistent symptoms and/or delayed or long-term complications. Therefore, apart from therapeutic protocols administered by physicians, there is a need to develop novel, efficacious, safe, and economically acceptable protocols to manage long term disease and treat related adverse events.

Probiotics are live microorganisms that are intended to have health benefits when consumed or applied to the human body with a proved efficacy in DGBIs. The popularity of using probiotics is growing and indications for their use are not limited to co-prescription with antibiotics only. Now more than ever, clinicians need clear guidelines and recommendations, including safety and efficacy for implementation of probiotics into clinical practice. Although there are no official recommendations on the use of probiotics in patients with COVID-19, we aimed to present the current knowledge and discuss clinical implications for the use of probiotics in post COVID-19 patients.

## 2. COVID-19 Pandemic and Microbiota

### 2.1. Environment

Common use of antiseptic and aseptic detergents and cleaning reagents in household, workplace, and public spaces can lead to a loss of microbial environmental diversity [8]. Commonly used detergents target the whole microbial environment and can facilitate horizontal antibiotic resistant genes among individuals [9]. According to “Hygiene Theory”, reducing the number of environmental microorganisms can diminish the risk of infection, however at the same time increase the risk of allergic disorders [9]. The current knowledge on the effect of cleaning detergents on the human microbiome is scarce and limited mostly to animal data. There is a constant need to expand our knowledge on the environmental effect and human health of the wide use of a variety of aseptic/prophylactic methods in the era of the COVID-19 pandemic [10]. The data are mounting that fermented food and probiotics are capable of diminishing the risk of environmental risk factors by limiting the microbial loss. Probiotics are aimed to maintain and support microbial biodiversity and limit the negative consequences of exposure to cleaning detergents [11]. However, this hypothesis has yet to be supported by the evidence-based medicine. Further studies in the field of environmental and evolutionary microbiology are needed in order to define recommendations for prebiotics and probiotics use to minimize microbiome alterations caused by environmental hygienization and implement their use into real life and practice.

### 2.2. Microbiota and COVID-19

The data are mounting that besides COVID-19 pulmonary infection, SARS-CoV-2 can also affect other organs, including the gastrointestinal tract. Gastrointestinal microbiota have been found to be altered in COVID-19 patients, who manifested with common GI symptoms. The presence of GI symptoms was associated with significantly increased risk of admittance to intensive care units (ICUs) and the rate of mortality in comparison to those without GI symptoms [12]. Individuals with comorbidities, such as diabetes mellitus (DM), hypertension, obesity, and cardiovascular diseases (CVD) and characterized by a low abundance of *Bacteroides* species were reported to have the highest COVID-19 mortality [13,14]. Hill et al. [15] in their pilot study of 15 patients with COVID-19 found persistent alterations in the fecal microbiome at a time of hospitalization, compared to control individuals, both of which were associated with fecal levels of SARS-CoV-2 and COVID-19 severity.

Microbiota alterations were reported as the risk factors for COVID-19 severity as assessed by univariate and multivariable logistic regression models. In contrast to mild COVID-19 patients, the gut microbiota of patients with moderate and severe symptoms have been found to be of: (a) lower Firmicutes/Bacteroidetes ratio; (b) higher abundance of *Proteobacteria*; and (c) lower abundance *Roseburia* and *Lachnospira.* It has also been demonstrated that the lower Shannon diversity index (odds ratio (OR) = 2.85, 95% CI = 1.09–7.41, *p* = 0.032) and higher (≥96.8 mg/L) C-reactive protein (OR = 3.45, 95% CI = 1.33–8.91, *p* = 0.011) were predictors of severe COVID-19 (a score of 6 or higher in the WHO Clinical Progression Scale). Others have also pointed out that hospitalized patients with moderate and severe COVID-19 disease had microbial signatures of gut dysbiosis and that the gut microbiota diversity has been pointed out as a prognostic biomarker of COVID-19 severity [16]. Moreover, Ward et al. [17] pointed that based on the composition of intestinal and oral microbiomes, scientists were able to predict, with more than 80% accuracy, the risk toward severe coronavirus disease and death.

Li et al. [18] using shotgun metagenomic sequencing and analyzing taxonomic indices found that the microbial diversity of COVID-19 patients was diminished as compared to controls. *Streptococcus thermophilus*, *Bacteroides oleiciplenus*, *Fusobacterium ulcerans*, and *Prevotella bivia* were found to be detected only in COVID-19 patients, whereas *Enterococcus faecium*, Candidate division TM7 single-cell isolate TM7c, *Actinomyces graevenitzii*, and *Solobacterium moorei* were found in healthy controls. In COVID-19 patients, nine metabolic pathways, among others D-glutamine and D-glutamate metabolism, thiamine metabolism, and pyruvate metabolism were significantly decreased, and several others, e.g., lysine degradation and arginine and proline metabolism increased as compared to healthy individuals. Overall, 15 species made up a set of microbiological markers of COVID-19. Moreover, the researchers found that some clinical indicators were associated with taxonomy. For instance, alanine transaminase (ALT), red blood cells (RBC), and hemoglobin levels correlated positively with *Coprococcus catus.* Other correlations were also found, including the asparagine transaminase (AST) and abundance of *Streptococcus salivarius*, *Clostridium nexile* counts correlated negatively with RBC, and hemoglobin but positively with neutrophils. The disease severity analysis among subgroups collectively confirmed a decreased number of species as compared to the healthy control, with two genera, namely, *Roseburia* and *Megasphaer*, negatively and *Paraprevotella*, *Lachnospiraceae*, and *Erysipelotrichaceae* positively linked with the severity of COVID-19. At the species level, six species including a butyrate producer *Roseburia inulinivorans*, were linked with the poor outcome in COVID-19 patients. On the other hand, *Paraprevotella* sp., *Streptococcus thermophilus*, *Clostridium ramosum*, and *B. animalis* showed the opposite trend in the association with the disease phenotype.

Of note, dysbiosis in patients with SARS-CoV-2 infection can be also caused by age, comorbidities, malnutrition, superinfections, antibiotics, and antivirals. Dysbiosis can affect intestinal, pulmonary, brain, and skin axes, which can be termed as “immunity dysregulation dysbiosis cycle” (IDDC) hypothesis in SARS-CoV-2 patients. Consequently, vicious circles of immune dysregulation, exaggerating, and leading to further microbiota alterations and dysbiosis followed by immune dysregulation may contribute to a severe prognosis and development of post-COVID-19 syndrome [19].

### 2.3. Post COVID-19 Syndrome

Post-acute COVID-19 syndrome (PACS) [7] as a phenotype of persistent symptoms and/or delayed or long-term complications beyond 4 weeks from the onset of symptoms [20], has been diagnosed in as much as 23.2% of COVID-19 patients. Approximately 10% of patients might have experienced health issues lasting up to a year long [21]. Commonly reported multisystem symptoms included: (i) fatigue, (ii) muscle weakness, and (iii) sleep difficulties [22]. The “loss of appetite”, nausea, vomiting, diarrhea, and abdominal pain make up a set of gastrointestinal symptoms [23]. These might occur as a consequence of SARS-CoV-2 ligation to the angiotensin-converting enzyme 2 (ACE2) receptors in intestinal epithelial cells, which might negatively affect normal gut function [24]. Importantly, nausea, abdominal pain, and diarrhea may last for 3 to 6 months after acute COVID-19 [25,26]. Multiple studies have shown that the taxonomy of gut microbiota may to at least some extent predict COVID-19 outcome [27,28,29]. Patients with an altered gut microbiota are more prone to develop severe forms of COVID-19 due to: (i) a higher risk of developing immunopathology linked to “leaky gut syndrome” [30], (ii) a higher risk of developing complications associated with the growth of opportunistic pathogens and the low counts of immunomodulatory beneficial bacteria [31]. Altered gut microbiota after the infection clearance [27,29] might be responsible for ongoing symptoms. For example, the overproduction of fermentation gas in the small intestine due to bacterial overgrowth might aggravate GI symptoms. [32]. On the other hand, beneficial microbes such as *Faecalibacterium prausnitzii*, *Eubacterium rectale*, and bifidobacteria were under-represented in symptomatic patients [27]. Interestingly, gut microbiota composition at hospital admission was linked to occurrence of post-acute COVID-19 symptoms (PACS). Patients without PACS showed a recovered gut microbiome profile at 6 months comparable to that of non-COVID-19 controls. A PACS-like microbiome were found to be abundant in *Ruminococcus gnavus*, *Bacteroides vulgatus*, and contained lower counts of *Faecalibacterium prausnitzii*. Persistent respiratory symptoms were associated with the presence of opportunistic gut pathogens, whereas neuropsychiatric symptoms were associated with nosocomial intestinal pathogens, such as *Clostridium innocuum* and *Actinomyces naeslundii* (all *p* < 0.05). Butyrate producers, such as *B. pseudocatenulatum* and *Faecalibacterium prausnitzii*, were inversely linked to PACS at 6 months. Further studies should formulate an opinion whether microbiota modulation can be somehow helpful in PACS [25].

Health care of patients with COVID-19 does not end with virus clearance ad PACS cases has been increasing along with new cases being discovered. Nutritional regimens to alleviate gastrointestinal symptoms might not only increase quality of life but also, to a lesser extent, important economic and social aspects, especially in the context of a long-term pandemic. [7].

Gastrointestinal dysbiosis after COVID-19 can occur, even in the absence of gastrointestinal symptoms [29]. In addition, antibiotic therapy, secondary bacterial infections, and enteral nutrition can also lead to dysbiosis [33,34]. Consequences of alterted microbiota can include inflammatory changes in the gastrointestinal tract, malnutrition [33], and viral and bacterial infections [29]. It is also possible that COVID-19 patients had an altered gut microbiota even before the disease [35]. In these patients, the COVID-19 may exacerbate dysbiosis leading to chronic diseases such as obesity and metabolic disorders [35]. It is important to consider that proper nutrition plays a significant role in restoring the microbiota after COVID-19 outbreeding, with prebiotics and probiotics playing a particular role [29,33]. By modulating the microbiota, we can expect to restore microbiome function and improve the immune response [36].

## 3. Probiotics and COVID-19

The modulation of the gut/lung microbiome with the implementation of probiotics is gaining new focus on the horizon [37], although the number of active or completed high level clinical trials is still low. Overall, these randomized controlled trials (RCTs) found that the ingestion of probiotics might favourably affect the outcome of COVID-19 with the most recently reported improvement regarding fatigue, anosmia and breathlessness, nausea, vomiting, and other gastrointestinal symptoms. On the other hand, probiotics should not be recommended to immunocompromised patients on corticosteroid therapy. Therefore, the use of prebiotics and postbiotics as well as next-generation probiotics or live immune-therapeutics may also represent a promising target of investigation in the fight against SARS-CoV-2 infection, as pointed out by Cunningham et al. [38].

### 3.1. Mechanism of Probiotics Action

**Figure 1:**
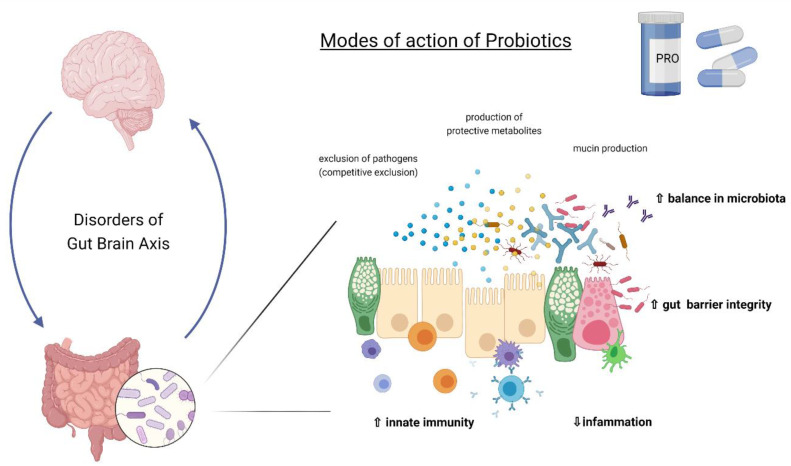
General mechanism of action of probiotics.

Due to their multidirectional action, probiotics can influence the course of COVID-19 infection through various mechanisms (Figure 1). These include: (i) improving anti-viral responses, (ii) production of antimicrobial peptides, (iii) protection from secondary infections, and (iv) antinflammatory activity [39]. Probiotics may have antiviral activity and modulate the immune response in the event of viral infections [40,41], and also reduce tissue damage resulting from infection [42,43]. Their direct effect on SARS-CoV-2 virus is associated with the production of antiviral compounds (hydrogen peroxide, nitric oxide, bacteriocins, subtilisin), inactivation of virulence factors, blocking of virus receptors, binding of its particles, direct and indirect blocking of the renin-angiotensin system, inhibition janus kinase (JAK)/signal transducer and activator of transcription protein (STAT) signaling pathway, and inhibition of histone deacetylase [44]. The immunomodulatory effects of probiotics is mainly based on increasing the production of IL-12, which activates NK, Th1, and Th2 immune cells, and increasing the production of IL-10 [45] leading to increased production of Treg cells to control inflammation [46,47,48]. A therapeutic approach with prebiotics, probiotics, and synbiotics can modulate other key points in the severity of COVID-19 cases: (a) reduced ferritin synthesis and regulation of iron metabolism by polyphenols (natural iron chelators), helping to diminish inflammation and oxidative stress [49,50]; (b) reduced D-dimer level involved in COVID-19 coagulopathy [51]; (c) increase in immune efficacy of COVID-19 vaccine [52,53]; and (d) reduced occurrence of persistent post-COVID-19 symptoms such as dyspnoea, tiredness, and joint and chest pain. The detailed description of mechanisms of probiotic action is beyond the scope of this paper and has been discussed elsewhere [54].

### 3.2. Clinical Efficacy

Several pilot clinical studies aimed at assessing the efficacy of probiotics in COVID-19 have already been published. For example, d’Ettorre et al. [55] administered *Streptococcus thermophilus* DSM 32345, *L. acidophilus* DSM 32241, *L. helveticus* DSM 32242, *Lacticaseibacillus paracasei* DSM 32243, *L. plantarum* DSM 32244, *LeviL. brevis* DSM 27961, *B. lactis* DSM 32246, and *B. lactis* DSM 32247 for seven days to 70 hospitalized patients with COVID-19. A reduction in diarrhea and other symptoms severity was shown in patients given probiotics compared to the placebo group. Such therapy was demonstrated to diminish the risk of respiratory diseases by eight times. In China, 800 COVID-positive patients experiencing diarrhea were supplemented with probiotics to finally recover from the disease faster including a relief in abdominal distension, nausea, vomiting, and other gastrointestinal symptoms [56]. Gutierrez-Castrellon et al. [57] performed a RCT involving 150 symptomatic COVID-19 outpatients that were treated with *L. plantarum* KABP022, KABP023 and KAPB033, and *Pediococcus acidilactici* KABP021 for 30 days. Remission rate of 53% and 28% in probiotic and placebo groups, respectively, were demonstrated. Tang et al. [58] recruited 1132 COVID-19-positive individuals and administered them with *L. rhamnosus* GG for 28 days but the results of the primary and secondary outcomes have yet to be published, although authors concluded that such option was inexpensive and safe. Finally, good clinical evidence exists to underline that probiotics improve human immunity, via inhibiting gut colonization by pathogens which consequently decrease the disease severity [59]. However, more studies have to be carried out to supporting the use of probiotics either in the form of oral supplements or nasal sprays as mitigating the effects of COVID-19.

### 3.3. Probiotics and Secondary Infections in COVID-19

SARS-CoV-2 virus infection is associated with secondary bacterial infections [60,61], especially in predisposing states such as, e.g., chronic obstructive pulmonary disease [62]. It might result from downregulation of immune genes [63]. Bacteria responsible for secondary infections predominantly are: *Escherichia coli*, *K. pneumoniae*, *P. aeruginosa*, *Methicillin-Resistant S. aureus* (MRSA) [64]. The basic procedure in the prevention of secondary infections is appropriate antibiotic therapy, however, some probiotics may find potential application here, especially those that have good adhesion properties to the gastrointestinal mucosa and antagonistic properties against pathogens. *L. plantarum* 299v adheres to epithelial cells along the entire length of the gastrointestinal tract in both healthy persons [65,66,67,68,69] and ill patients [70]. The binding of mannose residues exposed to the epithelial surface is the best-known mechanism allowing L. plantarum 299v to adhere to the intestinal epithelium [71]. *L. plantarum* 299v binds to epithelial receptors and competitively (through competition for receptors) inhibits the adhesion of numerous bacteria (f.i.: *Escherichia coli (ETEC/EPEC)*: *Salmonella enterica serovar Enteritidis*, *Vibrio cholerae*, *Pseudomonas aeruginosa* [72], *Streptococcus pneumoniae* [73], *Streptococcus aureus* [74], group A streptococci [75], staphylococci [76], and also *Candida albicans* [72,77] and *Schistosoma mansoni* [77]. Additionally, *L. plantarum* 299v increases the production of mucin by intestinal epithelial cells, which explains the antagonistic activity of this bacterium in relation to *Escherichia coli* [78,79].

### 3.4. Postbiotics

A panel of experts of International Scientific Association for Probiotics and Prebiotics (ISAPP) defined a postbiotic as a “preparation of inanimate microorganisms and/or their components that confers a health benefit on the host” [80]. Interest in postbiotics is still increasing due to their good stability, favourable safety profile, mechanism of action based on active molecules, beneficial effects on the intestinal microbiota, immune system, metabolism and signaling in the nervous system [80]. Few clinical trials and experimental studies regarding postbiotics and COVID-19 have been published.

A recent study reported that the metabolites of a *L. plantarum*, namely plantaricin BN, plantaricin D, plantaricin W, plantaricin JLA-9, blocked the protein S of SARS-CoV-2 [81]. Similar properties were presented with lactococcine G—another metabolite of *L. plantarum* [82].

### 3.5. Safety

Some probiotic strains can be responsible for bacteremia (e.g., *Lactobacillus strains*, *rhamnosus*, *acidophilus*, *casei* and *GG*; *Bacillus subtilis*; *B. longum* and *B. breve*), sepsis (*B. infantis*) and fungemia (i.e., *S. boulardii* and *S. cerevisiae*) in immunocompromised subjects, e.g., preterm infants.

Therefore, the high standard of quality control in production of probiotics is required (this problem is described in detail in Section 4) [83,84]. Other issues are: (i) contamination of probiotics with pathogenic bacteria, what could be especially harmful in vulnerable populations; (ii) wrong identification of strains; and (iii) loss of efficacy due to a decreased number of alive bacteria during storage period. Thus, authorities should be responsible for regulations concerning manufacturing of safe and effective probiotics [85,86,87]. Probiotics are Generally Recognised As Safe (GRAS) or Qualified Presumption of Safety (QPS), according to the Food and Drug Administration (FDA) and European Food Safety Authority (EFSA), respectively [88,89]. Despite many studies confirming the health benefits of probiotics, the health claims associated with the use of this product group have not yet been approved by the FDA and EFSA. The term prebiotics is also not officially recognised by the FDA and the European Union [87]. It is therefore necessary to establish a safe dosage and to resolve numerous complicated legislative issues [90]. It is also necessary to assess the benefit/risk ratio when using probiotics in immunocompromised patients, critically ill patients treated in an intensive care unit, and patients with a central vein catheter. Such situations are considered by some manufacturers to be a contraindication to the use of probiotics (e.g., *S. boulardii* CNCM I-745) [91]. The administration of probiotics via jejunostomy and the use of broad-spectrum antibiotics to which the probiotics used are resistant may also be risk factors [92]. Particularly noteworthy is the definition of the age categories in which the strains can be taken for specific indications. Of note, it is emphasized that the legislation or regulatory guidelines applied to prebiotics and probiotics vary significantly among countries, which in this time of the COVID-19 pandemic highlights the need to build globally uniform standards [93].

## 4. Probiotics—Quality Means Effectiveness

The legal regulations regarding the production of probiotics do not cover the specificity of this group of products and are the same as for food. In addition, the Food Safety Act practically excludes the possibility of informing consumers about the health effects of probiotics. Additional confusion is introduced by the division into probiotic drugs and dietary supplements. It often happens that the same strain, in the same quantity and produced by the same manufacturer, can be found both as a medicine and a dietary supplement. Therefore, medical practitioners are often confused and not eager to delve into these complex issues at a border of law and medicine, shifting the responsibility for the use of probiotics to patients or pharmacists. Probiotics must contain live bacteria, which are freeze-dried during production, i.e., dried at low temperature (this is the first critical moment for bacteria viability). Bacteria need to “animate” in the digestive tract and initiate the appropriate physiological and/or therapeutic effect. It is assumed that the minimum daily dose of probiotic bacteria should be approximately 1 × 10^9^ CFU (colony forming units) per day (deviations in the part of the logarithm are often not critical). Bacteria, after adhering to the epithelium of the gastrointestinal tract, transmit signals that “call” other microorganisms, which are to collectively create a biofilm that allows them to survive in the gastrointestinal tract. For this reason, it is more about the bacterial adhesion capacity than the dose of probiotic, which has the impact on the effectiveness of probiotics. The appropriate amount of probiotics in a given preparation should be kept at the end of the shelf-life. Other elements that ensure the viability of the bacteria throughout the shelf life of the product are the packaging that protects the probiotic from moisture (special barrier blisters are given out), transport under temperature-controlled conditions, and storage in a dry and cool place (some probiotics must be stored in the refrigerator). The most important is that probiotics must benefit the host’s health. That is, this effect should be documented by studies, preferably placebo-controlled clinical trials [94].

Probiotic strains must be well characterized, safe, and effective [94]. 

The most widely used technique for identifying strains is 16S ribosomal DNA sequencing. The sequence can be matched with available databases. The gold standard for strain identification, however, is whole genome sequencing (WGS), including any extrachromosomal elements. A fully sequenced genome allows for the recognition of microorganisms and facilitates the search for potential benefits and risk factors associated with the use of a given strain. One of the most important features of a safe probiotic is the lack of acquired antimicrobial resistance genes. Transmission of antibiotic resistance occurs through food, soil, and water reservoirs. That is why it is so important to properly select a probiotic strain, and ensure its verification and careful testing from every angle in order to prevent serious and irreversible consequences for the environment [94].

Assessing the viability of bacteria is one of the most important quality requirements for probiotics. Commonly used culturing methods are long-lasting, which do not ensure immediate correction of the production process and are burdened with the difficulties to culture various strains, which is especially important in the case of multi-strain products. The introduction of flow cytometry is very promising; however, it requires further validation studies [95].

**Figure 2:**
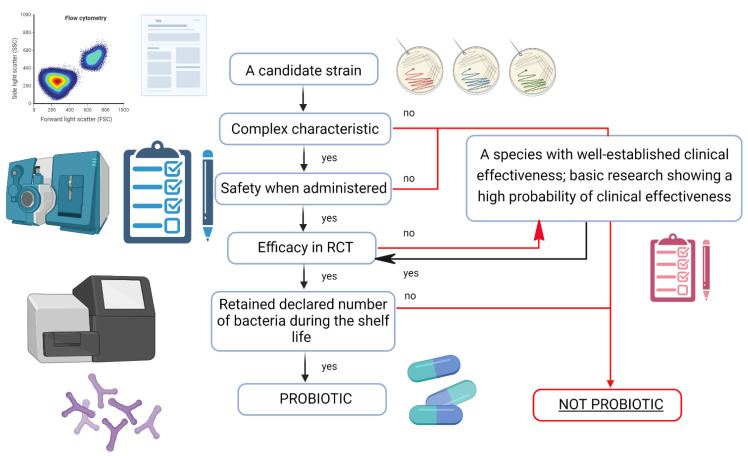
Decision tree for the classification of a strain as a probiotic (Adapted from [94]).

The safety of probiotic use must be determined based on established scientific principles, including appropriate research material. EFSA has been keeping and updating a list of species considered safe for human consumption since 2007. One of the basic classifications of probiotic strains is QPS. Qualification for QPS is made on the basis of taxonomic identification and comprehensive scientific data on the safety of a given strain. QPS qualification is conducted at species level. It seems that since the activity of probiotics is strain-dependent, the safety of their use should also be determined for individual strains. Now, the only method to evaluate this at a strain level is to conduct in vitro toxicological studies and clinical trials. End-product-specific tests are particularly important, especially in the case of using probiotics in groups of seriously ill people [96,97]. The decision tree for the classification of a strain as a probiotic is shown in Figure 2. 

Another problem concerns declaring the effectiveness of probiotics. The approach to this issue differs from country to country [98]. In the European Union, probiotics and dietary supplements are subject to food law (Regulation 178/2002/EC; Directive 2000/13/EU). All health claims made on probiotics must be approved by EFSA. EFSA has published a list of bacterial species with QPS. As already mentioned, EFSA has so far rejected all submitted health claims regarding probiotics. So, on the one hand, there is a stringent control of health claims, but on the other hand, there is little regulation of the manufacturing process and almost no post-marketing regulatory oversight. In the United States, most probiotic products are classified as food or dietary supplements. Dietary supplements must be manufactured in accordance with GMP guidelines, but are not covered by quality or efficacy testing. As in Europe, the packaging of dietary supplements cannot contain information regarding the indications for the use of probiotics in specific diseases, however, functional claims such as “supports healthy digestion” are allowed, with the reservation required by the FDA regarding the lack of medicinal properties of the product. These claims must be truthful, not mislead the consumer, and must be substantiated by scientific evidence. There is also a category of probiotics that has been developed to be ingested or administered enterally under medical supervision and which are intended for the dietary management of a specific disease or medical condition for which specific nutritional requirements exist. In the United States, these preparations are classified as medicinal foods. In Europe, manufacturers of dietary supplements cannot inform consumers about the effects of their products at all, unless they are accompanied by approved health claims. Therefore, the only way to obtain such information is to use professional studies or advice from doctors, pharmacists, nutritionists, and other health care system employees. Recommendations of scientific societies and other organizations setting standards of nutritional or dietary management are very important.

Taking into account the above considerations, below we discuss the evidence-based rationales of prescribing probiotics to post-acute COVID-19 patients, manifesting with gastrointestinal and neuropsychiatric symptoms. Despite lack of specific clinical trials among post COVID-19 patients with DGBIs, the available data already supports probiotic use in those patients.

## 5. Special Clinical Situations

### 5.1. COVID-19 and Central Nervous System

The body of evidence indicates that SARS-CoV-2 has the ability to invade the central nervous system (CNS) in either a direct way or—in most cases—indirectly, as a consequence of systemic hyper-inflammatory state. The viral agent was found to penetrate into brain parenchyma via at least a couple of routes [99]. It was hypothesized, however, that initially, peripheral nerves are invaded and then the infection spreads through synapses [100]. The olfactory nerve has been pointed as the most frequent route of infection [101] and seems a strong candidate as anosmia is a frequent COVID-19 symptom. Moreover, an angiotensin-converting enzyme 2 (ACE-2) receptors are co-expressed there with TMPRSS2 and neuropilins, among them NRP-1 invading CNS, all of which are necessary for SARS-CoV-2 infection [102,103]. Importantly, olfactory tract projections, for instance respiratory and cardiovascular control centers in medulla, have been found to contain SARS-CoV-2 [104], making the so-called transcribial route of neuroinfection. The other peripheral nerve found to play a role in SARS-CoV-2 infection is the vagus nerve, eventually reaching the brainstem [105]. Of note, the vagus nerve co-creates the gut brain axis, the dysfunction of which might explain neuropsychiatric symptoms that occur during COVID-19 [106]. SARS-CoV-2 was found in human faeces [107], as ACE receptors are expressed in intestinal epithelium. All together, gut originated viral particles might enter the enteric nervous system and finally reach the CNS via vagus nerve [108]. The other route of the virus neuroinvasion is of hematogenous type [109]. Viral transcytosis across vascular endothelial cells (expressing ACE-2 receptor) and the infected leukocytes mobilization towards the blood brain barrier make this one [110,111,112] the most probable and frequent one.

The first CNS-related symptoms might be due to hypoxia occurring along with respiratory distress. These alter the brain metabolism to a large extent [113] but do not serve as primary neurologic symptoms in COVID-19. Neuroinvasiveness and neurotropism are responsible for long COVID-19 symptoms. Short ones are nonspecific and include anosmia, lack of taste, headache, malaise, myalgias, and dizziness. Serious neurological disorders include acute disseminated encephalomyelitis (ADEM), such as demyelination, multiple sclerosis, optic neuritis, and encephalitis along with delirium and cerebrovascular events. Importantly, like in other pandemics in the past, psychiatric and cognitive sequelae do occur in COVID-19 survivors, despite the severity of the disease [114,115]. The mental well-being of each person has been altered by the pandemic-related stress to varying degrees, with children/adolescents, pregnant women, and the elderly being among the most affected [116]. It has been well established that extended exposure to overall stress contributes to alterations in biological stress-responsive systems (i.e., hypothalamic-pituitary-adrenal axis, autonomic nervous system, sympathetic-adreno-modulary system, immune system) and precipitate physical and mental disorders [117] or negatively affect ongoing conditions in people already affected. Behavioral restriction and social isolation also triggered unhealthy lifestyle choices which might additionally add on to poor neuropsychiatric state [118]. A large multinational survey, namely Collaborative Outcomes study on Health and Functioning during Infection Times (COH-FIT) [119,120] has been established in April 2020 to track the psychiatric state worldwide and to identify risk and protective factors towards poor outcomes. The very early results of the study, which up to the 1 January 2022 collected data on more than 166,000 respondents demonstrated worsening in stress, predominantly in women and the elderly. Similar observations were made for loneliness, anger, and obsessive-compulsive behaviors. These are in line with already published data stating that preventing mental health in long COVID-19 phenotypes might be of particular concern to health policy [121]. Indeed, the high prevalence of psychiatric disorders has become a catalyst for many unfavorable, social, health, and economic phenomena at the national and international level

### 5.2. COVID-19 and Gastrointestinal Tract

Up to date numerous retrospective and prospective studies as well as meta-analyses on the topic related to gastrointestinal manifestation of SARS-CoV-2 infection were published. Jin et al. [122] in their retrospective study reported at least one of common GI symptoms (nausea, vomiting or diarrhea) among 11.4% out of 651 patients due to SARS-CoV-2 infection at hospital admission in China. Most of the symptoms were self-limited; however, patients with gastrointestinal symptoms had more severe complications during treatment with a need of ICU admission [122] in comparison to those without gastrointestinal symptoms. In another multicenter, retrospective study and recruiting patients in China, investigators reported the presence of abdominal pain and melena in 21% of 230 COVID-19 patients [123]. Redd et al. in their cohort, multicenter retrospective study reported the presence of gastrointestinal symptoms (anorexia, diarrhea, nausea, and vomiting) in 61.3% out of 318 patients admitted to hospital due to SARS-CoV-2 infection [124]. Others also reported various frequency of GI symptoms among COVID-19 patients: (i) Cholaneril et al. [USA]—31.9% [125]; (ii) Zhang et al. [China]—39.6% [126]; and (iii) Cheung et al. [Hong Kong]—25.4% [127]. Li et al. in their meta-analysis, including 212 studies from 11 countries involving more than 280 000 individuals, reported that gastrointestinal (nausea, vomiting, abdominal pain) and respiratory symptoms (shortness of breath, chest pain) were associated with severe COVID-19, whereas pneumonia and end-organ failure were associated with mortality [128]. In another report, Li et al. reported no significant association between GI symptoms and COVID-19 mortality based on 20 studies with 58,423 COVID-19 patients with adjusted effect estimates analysis (pooled effect size, 0.93; 95% CI, 0.75 to 1.16; *p* = 0.535; random effects model) [129]. Cao et al. conducted a systematic review and meta-analysis of 31 studies with 46,959 individuals hospitalized in China and found the prevalence of diarrhea among 6.8% COVID-19 patients [130]. Another systematic review and meta-analysis of 47 papers and 10,890 patients revealed the presence of diarrhea in 7.7%, nausea and vomiting in 7.8%, and abdominal pain in 2.7% in patients hospitalized due to COVID-19 disease. Of importance, gastrointestinal symptoms were more prevalent in individuals hospitalized in countries other than China when comparison data analysis was conducted [131]. Transient liver function failure among COVID-19 individuals has also been observed and analyzed [4]. However, most acute gastrointestinal symptoms fade away, once the viral infection is cleared.

Nevertheless, SARS-CoV2 infection has already been implicated in persistence of chronic gastrointestinal symptoms, which may persist long after the COVID-19 disease [132]. SARS-CoV-2 has the ability to bind to ACE-2 receptors—found on the surface of many cells, including the gastrointestinal lining—and trigger their physiological role to processes conversion of angiotensin II (Ang II) to angiotensinI [133]. These two members of the RAAS family trigger opposing biological effects on target cells and stimulate: (i) the angiotensin 1 receptor (AT1R), and (ii) MasR, respectively. Activation of AT1R during SARS-CoV-2 infection has deleterious impacts, inducing an increase in reactive oxygen species (ROS) synthesis, vasoconstriction, fibrosis, and alterations of gut microbiota [134]. SARS-CoV-2, as capable of infecting gastrointestinal epithelial and endothelial cells, has been classified as an enteric pathogen. The interaction of the ACE2 receptor with the SARS-CoV-2 spike protein may trigger activation of the Nlrp3 inflammasome, which when hyperactivated further leads to cell death by pyroptosis [134]. Post-COVID-19 chronic gastrointestinal symptoms recall post-infectious irritable bowel syndrome (IBS), which is frequent after other viral and bacterial infections. According to ROME Foundation functional gastrointestinal disorders (FGIDs) or disorders of gut-brain interaction (DGBI) are disorders classified by GI symptoms related to any combination of: (i) motility disturbance, (ii) visceral hypersensitivity, (iii) altered mucosal and immune function; (iv) altered gut microbiota; and v) altered central nervous system (CNS) processing [135,136]. SARS-CoV-2 by altering mucosal, immune, and microbial function in the gastrointestinal tract impairs the gut barrier [136]. As we previously reported, an impaired gut barrier and microbial alterations may lead or be associated with gastrointestinal [54], mental [137], and metabolic [138] symptoms or disorders. Cooney et al. reported that gastrointestinal symptoms were common at 6 months among 43.8% individuals post COVID-19 disease [139]. Ours, yet unpublished data of a cohort of COVID-19 individuals in Poland, supports previous reports of a high percentage of patients with GI symptoms following COVID-19 recovery. Therefore, further research is essential to explore the nature of new post-COVID-19 symptoms as well as clinical courses and responses to various medical treatments.

### 5.3. Intensive Care of COVID-19 Patients—Evidence Based Medicine of a Single Center Experience

Here we share a brief report of a single center experience at the intensive care unit (ICU) at Pomeranian Medical University in Poland. ICU treatment significantly affected the COVID-19-related prognosis [3]. In one of the recent studies, Meconnen et al. reported a rate of 33% ICU transfer of hospitalized COVID-19 patients with a 33% mortality rate at ICU [140]. ICU treatment was associated with high mortality [141]. Variety of factors were reported to affect this outcome, including: (i) patients overall health condition at a time of hospitalization, (ii) age, (iii) comorbidities, and (iv) type of treatment. Most of the patients transferred to ICU were critically ill, with symptoms of acute respiratory failure due to viral pneumonia. Diagnostic x-ray and CT scans revealed matte glass appearance corresponding to massive pneumonitis affecting more than 90% of the lung area. Patients with corresponding lesions frequently required mechanical ventilation, deep analgo-sedation with muscle relaxant drugs administration and ECMO (Extra Corporeal Membrane Exygenation) along with other symptomatic pharmacological management. Despite the treatment, systemic hyper inflammatory state multiorgan failure affected the clinical picture. Targeted antibiotic therapy was frequently introduced to counteract secondary bacterial infections. Of recommended drugs, systemic steroids (dexamethason) were used in order to suppress systemic inflammation [142]. Of other ICU treatment regimens, anticoagulants in prophylactic (not therapeutic) doses were prescribed. Plasma of convalescents was not recommended to ICU critically ill patients. Similarly, the use of redemsivir was restricted only to patients within 72 h since COVID-19 diagnosis or as therapy continuation in patients transferring to ICU. (https://sccm.org/SurvivingSepsisCampaign/Guidelines/COVID-19, accessed on 30 July 2022).

Another problem reported among post-COVID ICU patients was significant undernutrition, which concerned 66% of patients (diagnosed based on Global Leadership Initiative on Malnutrition (GLIM) criteria). Despite intensive treatment, intensive catabolism was observed, which could have lasted for several months. Undernutrition was reported as an independent negative prognostic factor of ICU readmission, infection, and mortality. More than half of patients dismissed home after hospital and ICU treatment complained of poor health and incomplete recovery. Using the descriptive EQ5D-5L scale system, which comprises five dimensions: (i) mobility, (ii) self-care, (iii) usual activities, (iv) pain and discomfort, as well as (v) anxiety, complaints were more prevalent among women in their fifties [143]. Thromboembolic events were reported among other COVID-19-related disorders, including massive cerebral strokes. However, the full-scale epidemiologic data on thromboembolic events among COVID-19 patients are lacking. These thromboembolic events were reported also in young adults and children diagnosed with COVID-19 disease.

## 6. Specific Applications of Probiotics after COVID-19 Infection

### 6.1. Irritable Bowel Syndrome (IBS)

Disturbances in the interaction of the gut-brain axis play an important role in the multifactorial pathogenesis of the irritable bowel syndrome (IBS). The intestinal microbiota is an essential element of these interactions, and its disturbances directly affect other pathogenetic mechanisms of IBS. One of the first clinical trials assessing the effectiveness of probiotics in IBS, concerned the *L. plantarum* 299v strain in 40 patients with IBS. In all patients with IBS who took the probiotic for 4 weeks, a significant reduction in the frequency and intensity of abdominal pain was observed compared to the placebo group [144]. It should be noted that according to the current Rome IV Criteria, pain is a key symptom in IBS. In guidelines of Polish Gastroenterology Association [39], on the use of probiotic strains with a possible beneficial effect on the symptoms of IBS, experts—the authors of the monograph—suggested the use of individual strains or complex preparations tested for effectiveness in IBS rather than probiotics as a group. The recommended strains available in Poland include: *L. plantarum* 299V—1 × 10^10^ CFU 1 × daily, *Saccharomyces boulardii* CNCM I-745—5 × 10^9^ CFU 2 × daily, *Bacillus coagulans* GBI—15 × 10^7^ 3 × daily, and *B. infantis* 35624—10^8^ CFU 1 × daily. It should be emphasized that the recommendations are based on the results of randomized clinical trials (RCTs) and not meta-analyzes. The authors of the British guidelines in 2021, based on the RCT meta-analysis, indicated that probiotics should be used in persons with IBS in the first place, for 12 weeks with diet modification, before starting pharmacological treatment [145]. Although the authors of the guidelines do not recommend individual strains, in the meta-analysis attached to the guidelines, among the groups of probiotic bacteria, lactobacilli showed the greatest effectiveness in IBS. Marlicz et al. further analysis of the effectiveness of individual strains of lactobacilli in IBS showed the greatest effectiveness of *L. plantarum* 299v, the strain for which the largest number of RCTs were conducted [146]. These observations were also confirmed by McFarland et al. in the latest meta-analysis on the effectiveness of individual probiotic strains in patients with IBS [147]. The effectiveness of *L. plantarum* 299v was also confirmed in a large study, carried out in 25 medical centers in Germany, for a period of 12 weeks, comprising 221 patients with IBS. Taking the probiotic had a significant, beneficial effect on all the analyzed symptoms of IBS and was associated with the reduction in abdominal pain, flatulence, and normalization of the bowel movement rhythm [148]. Probiotic strains that can be used in patients with post-acute COVID-19 IBS are shown in Table 1.

### 6.2. Psychobiotics

The COVID-19 pandemic had an impact on almost every individual worldwide [154]. This refers not only to the infectious aspect of this clinical entity but also to global healhtcare crisis, including mental health among patients and their caregivers. Lockdowns, huge economic and social instabilities—all rose along the pandemic. A number of meta-analyses confirmed the high incidence of anxiety, depression, and PTSD in COVID-19 patients and frontline healthcare workers [155,156]. Pharmacotherapies are effective for some but many side effects might occur from COVID-19 and its link with gut microbiota urged the scientific and medical community to focus on the gut microbiome modulation as a novel strategy to counteract COVID-19-related symptoms, including neuropsychiatric ailments. As evidenced, stress increases gut permeability thus inducing peripheral and neuronal inflammation. Empirical data exist to confirm an important role of the gut microbiota in mental health and well-being; however, few gaps need to be addressed, with the causal role of the microbiota in mental health as the priority [157]. Secondly, the role of fungi and phages should also be evaluated, as these microorganisms also make up the microbiota. Importantly, a few conflicting results in human trials with respect to probiotics use in alleviating depression and anxiety exist [158], probably due to strain specificity. In addition, probiotics might not work in the same way in all individuals due to host genetic suits, one’s diet, and the colonization potential of the probiotics. Hence, one size might not fit all regarding anxiety and depression. Large trials in a diverse population to define efficacy, duration, adverse effects, and dosage are needed. Future trials might also include genetic determinants, for instance, those referring to probiotic colonization and their efficacy. In the era of post COVID-19 pandemic, personalized medicine is getting more and more attraction among scientists and healthcare providers. In particular, testing dietary interventions among individuals and testing their microbiomes might opt out as an effective strategy to boost well-being and mental health in patients recovering from COVID-19 disease. Probiotics as non-expensive and safe agents can be viewed as dietary supplements effective among individuals with risk factors, especially in developing countries being at high risk of depression or other stress-related comorbidities [159].

Psychobiotics, i.e., probiotics that support mental health, are currently of great interest to researchers, doctors, and patients suffering from mental disorders, as well as people exposed to stress. The pioneers of research on psychobiotics were John F. Cryan and Timothy G. Dinan, who introduced this term into the medical dictionary [160]. Animal studies provided the first evidence for the role of probiotics in shaping emotional health. It has been proved that the administration of psychobiotics influences the composition of the intestinal microbiota and may change the behavior of the tested animals [161,162,163]. In addition, it has been proven that selected probiotic strains, belonging to the group of psychobiotics, affect the activity of the vagus nerve, and if administered long enough, they can change the expression of GABA receptors related to the pathogenesis of anxiety-depressive disorders [164]. Moreover, numerous experimental and clinical studies have shown that some probiotics are able to lower the concentration of cortisol in humans [165] and its counterpart—corticosterone in animals [166]. It follows that they are also indirectly involved in the regulation of the hypothalamic-pituitary-adrenal (HPA) axis [137]. Disturbances in the functioning of this axis are related to the so-called allostatic load [167]. The term “allostasis” was coined to conceptualize the physiological responses that enable adaptation to environmental demands arising from stressful situations [168]. Allostasis mediators include hormones, neurotransmitters, neurotrophic substances, markers of oxidative stress, and inflammation. Short-term activation of these mechanisms may be beneficial to the process of maintaining homeostasis of the organism; however, chronic is a harmful burden on the organism and has been defined as an allostatic load (AL). In turn, the negative effects of this burden on mental and physical health have been defined as allostatic overload. These changes lead to mental disorders and chronic somatic diseases, such as metabolic or cardiovascular disorders. One of the factors influencing the allostatic load may be changes in the gut microbiota [169]. The results of recent years’ research clearly indicate that the use of psychobiotics may bring health benefits to patients treated for mental disorders. One of the best-studied strains of bacteria from this group are *L. heleveticus* Rosell-52 and *B. longum* Rosell-175. It has been shown that feeding *L. heleveticus* Rosell 52 to animals exposed to stress reduces the adhesion of pathogens to intestinal epithelial cells, prevents their translocation, and reduces the synthesis of pro-inflammatory cytokines and may have a protective effect on the structures of the limbic system exposed to long-term stress [170]. Interestingly, the administration of *L. heleveticus* Rosell-52 and B. longum Rosell-175 to mothers of rats that are subjected to high stress may save the next generations from the negative consequences of this burden [171]. Especially in the era of the latest research on the effects of stress from generation to generation, this observation gives great hope for effective help not only for directly burdened individuals but also for their offspring. Clinical studies have shown that the administration of *L. heleveticus* Rosell-52 and *B. longum* Rosell-175 bacterial strains to healthy people reduces gastrointestinal discomfort caused by excessive stress [172]. It has been observed that the administration of these bacterial strains has a positive effect on the well-being of the respondents, reduces the intensity of anxiety, and reduces the excretion of cortisol. In March 2016, Canada’s Natural and Nonprescription Health Products Directorate issued the following recommendations for its use: (1) it helps to relieve general symptoms of anxiety; (2) alleviate gastrointestinal symptoms caused by stress; which (3) supports emotional balance. Below we discuss issues related to psychobiotic use in patients with (i) depression, (ii) anxiety, (iii) and stress, what is especially important for patients after post-acute COVID-19 infection.

#### 6.2.1. Depression

In patients with depression, a different composition of the gut microbiota compared to healthy people was evidenced in multiple studies. The experimental animals, which have had stool transplanted from depressed patients, started to manifest depressive behavior after such a procedure. Although the mechanism of this relationship remains unknown [157], it is postulated that taxonomic changes observed in depressed patients are associated with pro-inflammatory effects, reduced production of short-chain fatty acids (SCFA), impaired intestinal barrier integrity, and disturbed metabolic pathways (inter alia tryptophan, GABA). However, only a few publications confirmed these via metagenomic and metabolomic analysis, evaluation of immunological parameters or markers of intestinal permeability. Future research requires a standardization process ranging from patient selection, biological material collection, sequencing (e.g., 16S, whole genome or “shotgun” shallow sequencing), and bioinformatics analysis. In clinical trials, the unequivocal effect of antidepressants on the microbiota is not observed, however, subsequent research projects require the involvement of a larger number of patients and mechanistic analyses [173,174]. It should be emphasized that the cause of depression is unknown, and the disease has a multifactorial etiology. Therefore, microbiota should not be treated as the Holy Grail that would answer all our questions regarding the etiology and treatment of this disease, but as an environmental element that participates in the etiopathogenesis of this disease and creates new possibilities for the monitoring and treatment of patients. Numerous studies and meta-analyses have shown the beneficial effect of probiotics on mood. Most of them were carried out in healthy people or with unconfirmed depression. A recently published systematic review comprised eight randomized clinical trials that investigated the relationship between the effectiveness of probiotics in depressed patients [175], and five of them also investigated the mechanism of action of probiotics. Other systematic reviews also describe studies of probiotics in patients with depression and other conditions, such as irritable bowel syndrome. Among the analyzed studies, four reported reductions in depression symptoms and two improved cognitive functions. Only one study in which probiotics were used as monotherapy did not confirm their clinical effectiveness. The composition of the gut microbiota was evaluated in one clinical trial and no changes were found under the influence of probiotics. It is however believed that probiotics, despite having no effect on the composition of the microbiota, affect the expression of bacterial genes and might induce anti-inflammatory responses within a cell. Two studies found a decrease in blood kynurenine. Additionally, an effect on the metabolism of tryptophan was observed after administration of the probiotic. Kynurenine may be of high importance in depressed patients due to its neurotoxic and neurodegenerative effects. Furthermore, it has recently been shown that the administration of *L. helveticus R0052* and *B. longum R0175* caused an increase in the concentration of Brain Derived Neurotrophic Factor (BDNF) in the blood, which may reduce the severity of depression symptoms [176]. Due to the scarcity of research, no clear conclusions can be drawn about the strains or their combinations, doses, and duration of their administration in depression; however, it is known that patients may benefit from the use of probiotics, especially in combination with antidepressants.

#### 6.2.2. Anxiety

The anxiolytic properties of probiotics have been observed in preclinical and clinical studies in which the influence of this group of preparations on the symptoms of anxiety accompanying other diseases, such as IBS, was assessed. So far, only one study has been published in which the impact of probiotics on generalized anxiety disorder (DSM-V criteria) in patients treated with sertraline has been analyzed. After eight weeks of the study, there was an improvement in the results of the Hamilton Rating Scale for Anxiety (HAM-A) [177].

#### 6.2.3. Stress

Stress-related comorbidities, such as depression and anxiety, were reported to be associated with gut microbiome alterations among COVID-19 frontline health care workers, persisting for at least half a year. During a half-year follow up, several microbes were linked to mental health status, including mainly *Faecalibacterium* spp. and [*Eubacterium*] eligens group spp. Both with anti-inflammatory effects. Of note, the prediction model indicated that low abundance of [*Eubacterium*] *hallii* group uncultured bacterium and high abundance of *Bacteroides eggerthii* at Day 0 (immediately after the two-month frontline work) were significant determinants of the reappearance of PTSD symptoms in FHWs [178]. Psychobiotic strains, which are useful in post-acute COVID-19 patients suffering from mental health problems, are included in Table 2.

## 7. Conclusions

COVID-19 pandemic had a significant impact on environmental, animal, and human microbiomes. Microbial alterations, frequently referred to as dysbiosis, played a role in the pathogenesis and prognosis of COVID-19 and associated diseases. Microbiota modulation by means of probiotics seems a very promising way of eliminating negative outcomes of SARS-CoV-2 infection. Particular probiotic strains have been tested and their efficacy documented in selected disorders, including IBS and neuropsychiatric disturbances. Currently, there are no official recommendations or recommended procedures for the use of probiotics in patients with COVID-19. However, it can be assumed that many doctors, pharmacists, and patients will want to use a probiotic in the treatment of this disease. In such cases, strains with documented activity should be used. There is a constant need to plan and conduct new RCTs on the role of probiotics and verify their clinical efficacy of counteracting negative consequences of COVID-19 pandemic. Quality control is another important but often neglected aspect in trials utilizing probiotics in various clinical entities. It determines the safety and efficacy of probiotics, which is of utmost importance in patients with post-acute COVID-19 syndrome.

## Figures and Tables

**Table 1:** Probiotic strains potentially useful in post-COVID-19 IBS [146,149,150,151,152,153].

Strains	Daily Dose (CFU)
*L. plantarum* 299v (DSM 9843)	1 × 10^9^–2 × 10^10^
*B. infantis* 35624	1 × 10^8^
*S. boulardii* CNCM I-745	2 × (5 × 10^9^)/or 250 mg–4 × 10^11^
*E. coli* DSM17252	3.375 × 10^7^–2.475 × 10^8^
*Bacillus coagulans* GB-1-30, 6068	2 × 10^9^
*S. cerevisiae*	4 × 10^9^
*B. lactis* DN 173010	2 × 125 g
*L. rhamnosus* NCIMB 30174, *L. plantarum* NCIMB 30173, *L. acidophilus* NCIMB 30175, and *Enterococcus faecium* NCIMB 30176	2 × 10^7^/kg body weight
*S. thermophilus* DSM24731, *B. longum* DSM24736, *B. breve* DSM24732, *B. infantis* DSM24737, *L. acidophilus* DSM24735, *L. plantarum* DSM24730, *L. paracasei* DSM24733 and *L. delbrueckii* spp. *bulgaricus* DSM24734	4.5–9.0 × 10^11^
*L. aciophilus CUL60* [NCIMB 30157], *L. acidophilus CUL21* [NCIMB 30156], *B. animalis* subsp. *lactis CUL34* [NCIMB 30172] and *B. bifidum CUL20*	2.5 × 10^10^
*L. plantarum CECT7484* and *CECT7485* and *Pediococcus acidilactici CECT7483*	3.6 × 10^9^
*L.* sp. *HY7801*, *B. longum HY8004* and *L. brevis HY7401*	4 × 10^10^
*B. bifidum BGN4*, *B. lactis AD011*, *L. acidophilus AD031* and *L. casei IBS041*	4 × 10^10^
*B. lactis CNCM I-2494*, with *S. thermophilus* and *L. bulgaricus*	1.25 × 10^10^ + 1.2 × 10^9^

**Table 2:** Psychobiotics strains potentially useful in post COVID-19 mental problems.

Strains	Daily Dose (CFU)	Ref.
Stress		
*L. acidophilus* Rosell-52; *B. longum* Rosell-175	3 × 10^9^	[172]
*L. casei Shirota* YIT 9029	1 × 10^10^	[179]
*L. plantarum* DR7	1 × 10^9^	[180]
Anxiety and/or depressive symptoms		
*L. casei* W56, *L. acidophilus* W22, *L. paracasei* W20, *B. lactis* W51, *L. salivarius* W24, *Lactococcus lactis* W19, *B. lactis* W52, *L. plantarum* W62 and *B. bifidum* W23	7.5 × 10^6^	[181]
*L. casei Shirota*	2.4 × 10^10^–4.2 × 10^11^	[182,183]
*L. helveticus* R0052, *B. longum* R0175	3 × 10^9^	[165,184]
*B. lactis* W52, *L. brevis* W63, *L. casei* W56, *Lactococcus lactis* W19 *and* W58, *L. acidophilus* W37, *B. bifidum* W23, *B. lactis* W51, *L. salivarius* W24	5 × 10^9^	[185]
Major Depressive Disorder (MDD)		
*L. plantarum* 299v	2 × 10^10^	[186]
*L. helveticus* R0052 (CNCM strain I-1722) and *B. longum* R0175 (CNCM strain I-3470)	1 × 10^10^	[187,188]
*L. casei*, *L. acidofilus*, *L. bulgarigus*, *L. rhamnosus*, *B. breve*, *B. longum*, *S. thermophilus* and FOS	*L. casei* 3 × 10^8^; *L. acidofilus* 2 × 10^8^, *L. bulgarigus* 2 × 10^9^, *L. rhamnosus* 3 × 10^8^, *B. breve* 2 × 10^8^, *B. longum* 1 × 10^9^, *S. thermophilus* 3 × 10^8^ and 200 mg fructooligosaccharide	[189]
*B. bifidum* W23, *B. lactis* W51 and W52, *L. acidophilus* W37, *L. brevis* W63, *L.casei* W56, *L. salivarius* W24, *L. lactis* W19 W58	1 × 10^10^	[190]
*L. acidophilus*, *L. casei* and *B. bifidum*	1 capsule daily	[191]
*L. plantarum* Heal 9 + SAMe	1 × 109 + 200 mg SAMe	[192]
*B. bifidum* W23, *B. lactis* W51, and W52, *L. acidophilus* W22, *L. casei* W56, *L. paracasei* W20, *L. plantarum* W62, *L. salivarius* W24, *L. lactis* W19 and FOS	7.5 × 10^9^	[193,194]

SAMe—Sadenosylmethionie; FOS—fructooligosaccharides.

## Data Availability

Not applicable.
